# Efficacy and safety of an innovative prolonged-release combination drug in patients with distal renal tubular acidosis: an open-label comparative trial versus standard of care treatments

**DOI:** 10.1007/s00467-020-04693-2

**Published:** 2020-07-26

**Authors:** Aurélia Bertholet-Thomas, Catherine Guittet, Maria A. Manso-Silván, Arnaud Castang, Véronique Baudouin, Mathilde Cailliez, Massimo Di Maio, Olivia Gillion-Boyer, Emilija Golubovic, Jérôme Harambat, Alexandre Klein, Bertrand Knebelmann, François Nobili, Robert Novo, Ludmila Podracka, Gwenaëlle Roussey-Kesler, Christos Stylianou, Luc-André Granier

**Affiliations:** 1 Centre de Référence des Maladies Rénales Rares – Néphrogones – Hôpital Femme Mère Enfant – Filière ORKiD, Hospices Civils de Lyon, Bron, France; 2 Advicenne, Nîmes, France; 3 Service de Néphrologie Pédiatrique, Hôpital Robert Debré, Paris, France; 4 Service de Pédiatrie Multidisciplinaire, Hôpital de la Timone, AP-HM, Marseille, France; 5 Service de Réanimation Néonatale et Néonatologie, CHU de Nîmes, Nîmes, France; 6 Service de Néphrologie Pédiatrique, Centre de Référence des Maladies Rénales Héréditaires de l’Enfant et de l’Adulte (MARHEA), Institut Imagine, Hôpital Necker-Enfants Malades, Université de Paris, Paris, France; 7 Klinički Centar Niš, Klinika za dečije interne bolesti – Odeljenje za nefrologiju, Niš, Serbia; 8 Service de Pédiatrie, CHU de Bordeaux, Hôpital Pellegrin-Enfants, Bordeaux, France; 9 Service de Néphrologie, Pôle DIACOR, Hôpitaux Civils de Colmar, Colmar, France; 10 Service de Néphrologie adultes Hôpital Necker, Paris, France; 11 Service de Pédiatrie 2, Hôpital Jean Minjoz, CHU de Besançon, Besançon, France; 12 Service de Néphrologie Pédiatrique, Hôpital Jeanne de Flandre, CHRU de Lille, Lille, France; 13 Department of Pediatrics, National Institute of Children’s Health, Bratislava, Slovakia; 14 Unité de Néphrologie et Hémodialyse Pédiatrique, Clinique Médicale Pédiatrique Hôpital Mère-Enfant, CHU de Nantes, Nantes, France; 15 ClinBay Ltd., Limassol, Cyprus

**Keywords:** dRTA, Plasma bicarbonate, Plasma potassium, Palatability, Gastrointestinal tolerability

## Abstract

**Background:**

Distal renal tubular acidosis (dRTA), due to impaired acid secretion in the urine, can lead to severe long-term consequences. Standard of care (SoC) oral alkalizers, requiring several daily intakes, are currently used to restore normal plasma bicarbonate levels. A new prolonged-release formulation, ADV7103, has been developed to achieve a sustained effect with an improved dosing scheme.

**Methods:**

In a multicenter, open-label, non-inferiority trial (*n* = 37), patients with dRTA were switched from SoC to ADV7103. Mean plasma bicarbonate values and proportion of responders during steady state therapy with both treatments were compared, as were other blood and urine parameters, as well as acceptability, tolerability, and safety.

**Results:**

When switching from SoC to ADV7103, the number of daily intakes was reduced from a median of three to twice daily. Mean plasma bicarbonate was increased and non-inferiority of ADV7103 was demonstrated (*p* < 0.0001, per protocol), as was statistical superiority (*p* = 0.0008, intention to treat [ITT]), and the response rate increased from 43 to 90% with ADV7103 (*p* < 0.001, ITT). Urine calcium/citrate ratio was reduced below the threshold for risk of lithogenesis with ADV7103 in 56% of previously non-responders with SoC (*p* = 0.021, ITT). Palatability was improved (difference [95% CI] of 25 [10.7, 39.2] mm) and gastrointestinal discomfort was reduced (difference [95% CI] of − 14.2 [− 25.9, − 2.6] mm) with ADV7103.

**Conclusions:**

Plasma bicarbonate levels and response rate were significantly higher with ADV7103 than with SoC. Urine calcium/citrate ratio, palatability, and gastrointestinal safety were significantly improved, supporting the use of ADV7103 as first-line treatment for dRTA.

**Trial registration:**

Registered as EudraCT 2013-002988-25 on the 1st July 2013

**Figure:**
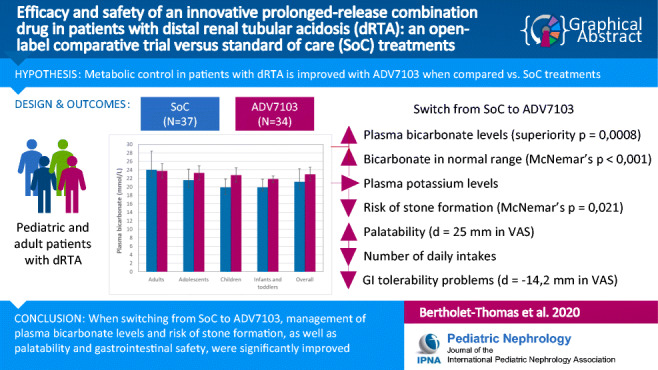
Graphical abstract

**Electronic supplementary material:**

The online version of this article (10.1007/s00467-020-04693-2) contains supplementary material, which is available to authorized users.

## Introduction

Distal renal tubular acidosis (dRTA) is a rare disorder due to an impaired net secretion of acid by the distal tubule, resulting in hyperchloremic metabolic acidosis, often combined with hypokalemia as a result of renal potassium wasting [1–3]. Distal RTA arises when transporters or transcription factors implicated in renal proton secretion are genetically altered [4–6] or affected as a consequence of an autoimmune disease [7–9].

Failure to thrive is the most common presentation of dRTA in infants and children [10]. If not adequately treated, dRTA causes great damage to bone and kidney [2, 11–15]. In some cases, particularly when associated to an autoimmune disease, dRTA may also present as a metabolic emergency, including manifestations such as hypokalemic paralysis, metabolic coma, and, in extreme cases, death [7, 10].

The standard of care (SoC) therapy consists of administration of oral alkalizing agents, usually as immediate-release bicarbonate and/or citrate salts, and potassium supplements if required [2, 4, 13, 16]. Drug doses need to be adjusted to maintain normal plasma bicarbonate concentrations [17].

Current treatments are characterized by poor efficacy, inconvenient dosing schemes requiring multiple day and night administrations, gastrointestinal discomfort, bad taste, and poor adherence, with only half of patients achieving correct metabolic control according to a recent report in patients with primary dRTA [11].

ADV7103 is an innovative, prolonged-release oral granule formulation (2 mm tablets) combining the advantages of potassium citrate and potassium bicarbonate [18]. It has been developed with the aim to improve the absorption profile of the alkalizing agents, delivering sustained efficacy over 12 h, and thus reducing administration to only two intakes per day.

The aim of the study was to evaluate short-term efficacy, acceptability, tolerability, and safety of ADV7103 in comparison to current SoC treatments in adult and pediatric patients with dRTA.

## Materials and methods

### Study population and design

A multicenter, open-label, non-randomized, non-inferiority phase II/III trial (EudraCT 2013-002988-25), in which adult and pediatric patients with dRTA were switched from SoC to ADV7103 treatment, was performed according to a protocol discussed with the European Medicines Agency (EMA), considering the small size of the target population.

During the study, male and female patients (aged 6 months to 55 years old), with acquired or inherited forms of dRTA, were enrolled.

Patients were excluded from the study based on the following criteria: unusual additional proximal tubular signs, hyperkalemia (plasma potassium > 5.0 mmol/L), moderate or severe kidney impairment (GFR < 45 mL/min/1.73 m^2^), or any other condition that could be negatively affected by the study medication or that could affect the study medication. Patients receiving potassium-sparing diuretics, angiotensin-converting enzyme inhibitors, angiotensin II receptor antagonists, or tacrolimus were also ruled out.

Enrolment was performed according to a staggered approach, where review of safety/tolerability data of older patients completing the study by a Data Safety Monitoring Board allowed validation of subsequent enrolment of younger patients.

**Fig. 1:**
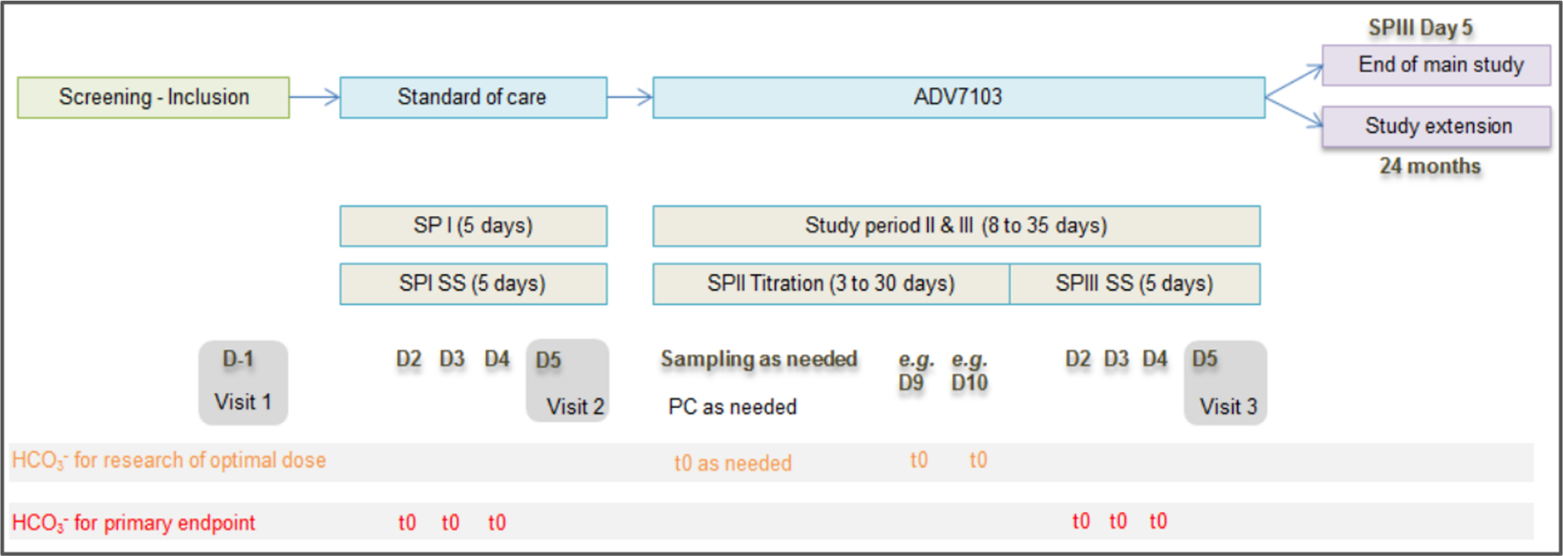
Design of the study. *D* day, *h* hour, *PC* phone call, *SP* study period, *SS* steady state, *t*0 timepoint before first morning dose

Patients participated for three consecutive study periods (Fig. 1):

- Period I: Five-day steady state treatment with SoC and any potassium supplements respecting usual therapeutic dose and dosing schemes.
- Period II: Titration phase aimed at determining the therapeutic ADV7103 dose.
- Period III: Five-day steady state treatment with ADV7103 at the therapeutic dose.

For titration of ADV7103 during period II, dose increments of 0.5, 1.0, or 1.5 mEq/kg/day were considered, without exceeding 10 mEq/kg/day.

### Plasma bicarbonate and plasma potassium

The primary endpoint was the mean pre-morning dose level of plasma bicarbonate, over three consecutive days (day 2, day 3, and day 4) on steady state therapy (see Fig. 1). Patients were considered non-responders when their mean plasma bicarbonate concentration was below the lower normal value of the local laboratory. As a secondary evaluation, patients were considered non-responders when any single value of plasma bicarbonate was below the lower normal value, as an indicator of the robustness of metabolic control.

Plasma potassium was evaluated using the same blood samples, as well as samples drawn during day 5 (Fig. 1). Patients were considered as hypokalemic when any single value was below the normal range of the local laboratory.

Detailed information about the methods used for plasma bicarbonate and plasma potassium and the normal ranges defined by each local laboratory is provided as supplementary information.

### Urine parameters

Urine parameters were determined during steady state with SoC and with ADV7103 on urine samples collected before treatment administration on day 4 and day 5 (Fig. 1).

Calcium and citrate to creatinine ratios were calculated and expressed in mmol/mmol. Hypercalciuria was considered when patients presented at least one episode of the urine calcium to creatinine ratio (UCa/UCr) above the normal values previously defined according to age and gender [19, 20]. Hypocitraturia was considered when patients presented at least one episode of the urine citrate to creatinine ratio (UCi/UCr) below the normal values found in the literature according to age and gender [21].

Increased risk of stone formation was considered in patients presenting at least one episode of the urine calcium to citrate ratio (UCa/UCi) above the reported threshold to evaluate the risk of lithogenesis of 3 mmol/mmol [22]. Although this index is not as relevant in young children as in older children and adults, it was considered to indicate a more equilibrated ratio between calcium and citrate excretion.

Detailed information about the normal values used for urine parameters is provided as supplementary information.

### Palatability and acceptability

Palatability was evaluated by the end of both steady state treatment periods using a self-rated 100 mm visual analogue scale (VAS) in adults and adolescents (score of 0 meaning “dislike very much” and a score of 100 “like very much”), an equivalent 100 mm parent-rated VAS in children < 4 years of age, and a validated self-rated 5-point facial hedonic scale (FHS) in children 4–11 years of age (with the support of their parents if required). FHS scores were converted to VAS scores (and vice-versa) so that all patients could be assessed together despite the differences in the evaluation methods. Additionally, scores for ease of administration and ease of swallowing were evaluated similarly with appropriate self-rated or parent-rated 100-mm VAS scales (a score of 0 meaning “very difficult” and a score of 100 “very easy”).

### Safety and tolerability

Adverse events (AEs) were recorded and graded according to their severity and causal relationship with the treatments. Any abnormal values on blood or urine parameters, physical examination, and vital signs were also recorded. Hyperkalemia was specifically followed.

Gastrointestinal tolerability was evaluated at the end of both steady state treatment periods with appropriate (self-rated or parent-rated) 100 mm VAS, except for children 4–11 years of age, for whom a validated self-rated 5-point FHS was used. FHS scores were converted to VAS scores (and vice-versa).

### Statistical methods

All patients with at least one efficacy assessment were included in the intent-to-treat (ITT) analysis set. All patients who completed the study with no major protocol deviations impacting on efficacy were included in the per protocol (PP) analysis set.

Missing plasma bicarbonate values for calculation of the primary endpoint were replaced by additional values obtained on day 1 or day 5 of the same period, as long as they were quantified under exactly the same conditions (i.e., before morning dose, same laboratory, same analysis method, same equipment, and same normal ranges). The mean value was calculated when at least one of the three values was available.

Individual mean values of plasma bicarbonate obtained with ADV7103 and SoC were first compared in a non-inferiority analysis in the PP set. At least 24 evaluable patients were required to have at least 80% power for the primary non-inferiority assessment, assuming a one-sided paired *t* test at the 2.5% significance level, with a standard deviation of 4.1 mmol/L and a non-inferiority margin of − 2.5 mmol/L. Non-inferiority of ADV7103 was considered when the lower, one-sided 97.5% of the mean difference between ADV7103 and SoC lay entirely on the positive side of the non-inferiority margin.

A mixed-effects analysis of variance (ANOVA) in the ITT set, which included the treatment as a fixed effect and the patient as a random effect, was used to assess superiority. This was done after non-inferiority was declared, in line with the EMA and the FDA recommendations [23, 24]. Values of least squares (LS) mean, standard error (SE), and two-sided 95% confidence interval (CI) were reported for the difference between ADV7103 and SoC.

Proportions of non-responders and responders were reported in contingency tables for the different blood and urine parameters evaluated with SoC and ADV7103. In order to compare both treatments, McNemar’s test was used (significance level α = 5%). If the *p* value was significant for a given parameter, the null hypothesis of homogeneity of the proportions was rejected.

The number/proportion of patients presenting AEs, incidence, and severity of these AEs and incidence of abnormal values on safety laboratory parameters, physical examination, and vital signs were reported. Descriptive analyses were performed for each treatment, overall and by age group in the safety analysis (SA) set (patients who received at least one dose of any of the study treatments).

The analysis of gastrointestinal tolerability and treatment acceptability was performed using a mixed model with treatment as a fixed factor and an unstructured covariance matrix for the repeated measures by patient and LS mean, SE and two-sided 95% CI values were reported for the difference between treatments in the acceptability analysis (AA) set (patients who received at least one dose of any of the study treatments and with at least one acceptability assessment).

## Results

A total of 37 patients (36 with inherited dRTA and 1 with dRTA as a consequence of Sjögren’s disease) were enrolled in 13 different centers in France, Serbia, and Slovakia. The study took place between September 2014 and May 2016. Patient enrolment/completion and population characteristics are summarized in Table 1. Five (13.5%) patients dropped out prematurely (a child due to the high number of blood tests needed, an infant, a toddler, an adolescent due to problems in taking ADV7103 treatment, and an adolescent due to lack of efficacy with ADV7103) and 32 patients completed the three study periods.

**Table 1:** Patient disposition and summary of demographic data by age group and overall

	Adults ≥ 18 years	Adolescents 12–17 years	Children 4–11 years	Infants and toddlers 0.5–3 years	Overall
*n* patients enrolled	7	10	15	5	37
*n*(%) females	5 (71%)	8 (80%)	9 (60%)	1 (20%)	23 (62%)
*n*(%) males	2 (29%)	2 (20%)	6 (40%)	4 (80%)	14 (38%)
Age (years)					
Mean (SD)	23.3 (9.9)	14.0 (1.7)	7.3 (2.4)	2.6 (1.1)	11.5 (8.2)
Median (range)	19.3 (19–46)	13.6 (12–17)	7.4 (5–12)	3.0 (1–4)	11.5 (1–46)
Weight (kg)					
Mean (SD)	69.1 (22.6)	43.7 (7.6)	26.5 (12.5)	13.4 (3.8)	37.4 (22.3)
Median (range)	60.5 (51–114)	41.9 (32–57)	23.3 (12–54)	12.5 (9–19)	39.0 (9–114)
Height (cm)					
Mean (SD)	160.3 (7.5)	156.6 (10.0)	119.7 (16.5)	90.9 (11.1)	133.5 (27.8)
Median (range)	164 (149–168)	157 (139–170)	117 (91–154)	94 (75–102)	139 (75–170)
BMI (kg/m^2^)					
Mean (SD)	26.6 (7.1)	17.8 (2.6)	17.5 (3.7)	16.0 (1.4)	19.1 (5.4)
Median (range)	23.8 (20–41)	16.7 (15–23)	15.9 (13–24)	15.9 (14–18)	16.8 (13–41)
*n* patients SPI completed	7	10	14	4	35
*n* patients SPIII completed	7	8	14	3	32
*n* drop-outs	0	2	1	2	5

*BMI* body mass index, *SD* standard deviation, *SP* study period

### Description of treatments

A great diversity of SoC products in the form of immediate-release formulations was observed. Almost half of the patients (48.6%) took more than one alkalizing product. Three patients (8.1%) received potassium chloride as a supplement to their SoC treatment. A large proportion of patients (62.2%) were receiving sodium salts.

The number of daily intakes with SoC ranged between 3 and 6 in 32 patients (86.5%) and 10 patients (27%) had to take their medication during night-time, which was particularly frequent in children. Mean ± SD alkali doses prescribed with SoC were 1.99 ± 1.54, 2.20 ± 1.41, 2.70 ± 1.23, and 5.27 ± 2.54 mEq/kg/day, in adults, adolescents, children, and infants/toddlers, respectively.

In contrast, only two intakes of ADV7103 (morning and evening) were given. The mean ± SD alkali doses prescribed with ADV7103 were 1.74 ± 1.05, 2.79 ± 1.74, 3.80 ± 1.15, and 6.11 ± 2.26 mEq/kg/day, in adults, adolescents, children, and infants/toddlers), respectively.

### Plasma bicarbonate levels

**Fig. 2:**
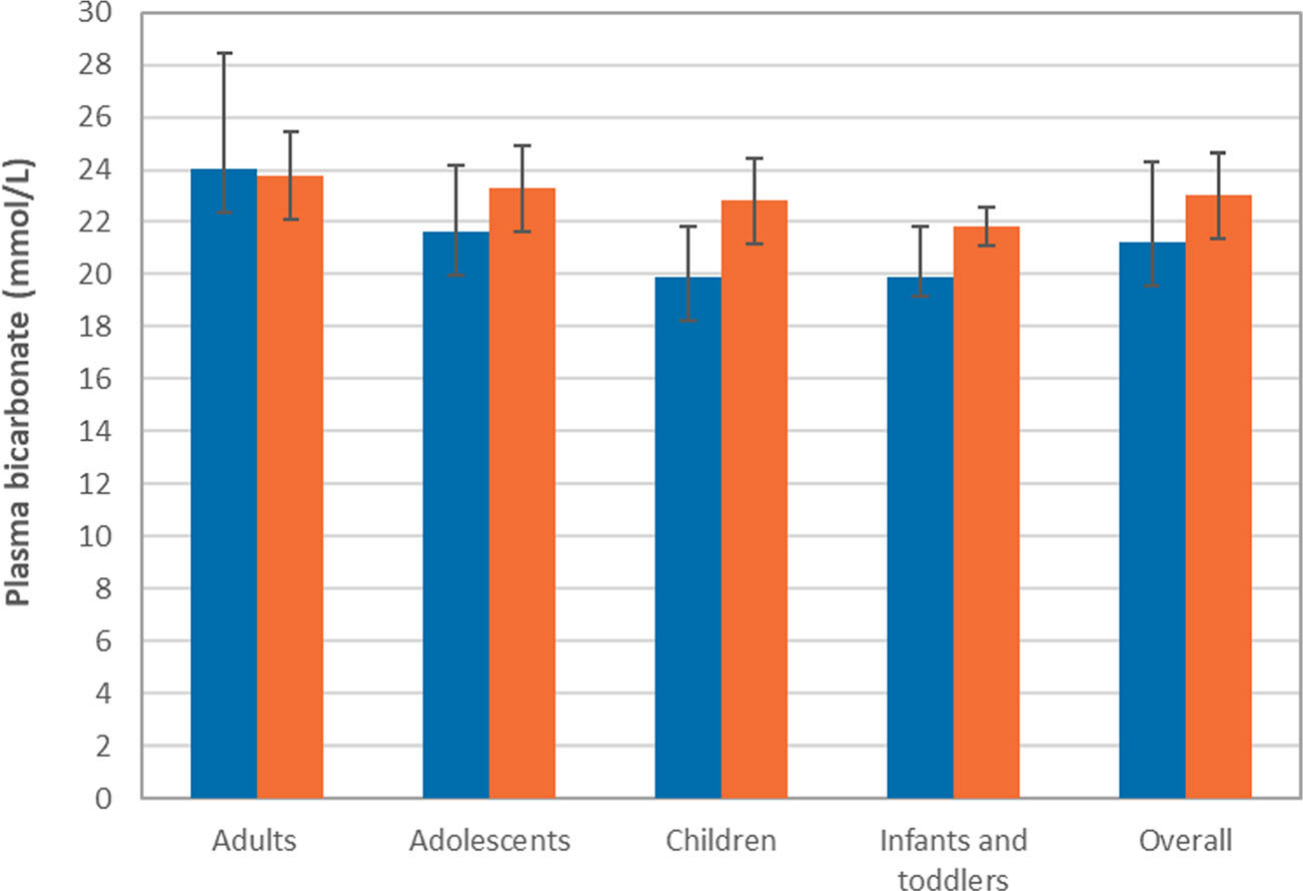
Steady state mean (± SD) plasma bicarbonate levels in the different age groups before administration of the first morning dose of SoC (multiple daily intakes) and ADV7103 (morning and evening), ITT set. Blue bars: SoC, orange bars: ADV7103

An improvement of plasma bicarbonate levels and less variability were observed with ADV7103 compared to SoC (Fig. 2). The overall mean ± SD plasma bicarbonate values were 21.7 ± 3.1 and 23.1 ± 1.6 mmol/L, respectively, with SoC (*n* = 29) and ADV7103 (*n* = 30) considering the PP set, and 21.2 ± 3.1 and 23.0 ± 1.6 mmol/L, respectively, with SoC (*n* = 34) and ADV7103 (*n* = 31) considering the ITT set.

As the non-inferiority of ADV7103 vs. SoC was demonstrated considering the mean plasma bicarbonate values (difference (95% CI) of 1.42 (0.41, 2.43), *p* < 0.0001, PP set), a subsequent superiority analysis was performed, also showing a statistically significant difference in favor of ADV7103 (LS mean difference (95% CI) of 1.64 (0.67, 2.60), *p* = 0.0008, ITT set).

Non-responder/responder analyses for mean plasma bicarbonate values considering the ITT set (*n* = 30) indicated that only 13 (43.3%) patients treated with SoC presented mean plasma bicarbonate values above the lower normal value, while 27 (90%) had normal values with ADV7103. A total of 14 (82.4%) patients from the 17 non-responders with SoC became responders when switching to ADV7103, while among the 3 non-responders with ADV7103, none was previously responder with SoC (Table 2). The difference in the proportions of responders/non-responders between both treatments was statistically significant (McNemar’s *p* < 0.001, ITT set).

**Table 2:** Contingency tables showing the number (%) of responders for plasma bicarbonate values and non-responders, presenting, respectively, mean plasma bicarbonate values (mmol/L) below the normal lower limit, and at least one value of plasma bicarbonate values (mmol/L) below the normal lower limit, with SoC and ADV7103

Mean plasma bicarbonate (ITT set, *n* = 30)		ADV7103	
		R	NR
SoC	R	13 (43%)	0 (0%)
	NR	14 (47%)	3 (10%)
*p* value		< 0.001*	
Plasma bicarbonate (ITT set, *n* = 30)		ADV7103	
		R	NR
SoC	R	10 (33%)	1 (3.3%)
	NR	13 (43%)	6 (20%)
*p* value		0.002*	

*NR* non responders, *R* responders

* Significant difference (according to McNemar’s test)

In the particular case of the adult female presenting an acquired form of dRTA (due to Sjögren’s disease), mean plasma bicarbonate levels were in the normal range with both SoC and ADV7103 (22.7 ± 1.5 and 22.7 ± 0.6 mmol/L, respectively).

### Plasma potassium levels

**Fig. 3:**
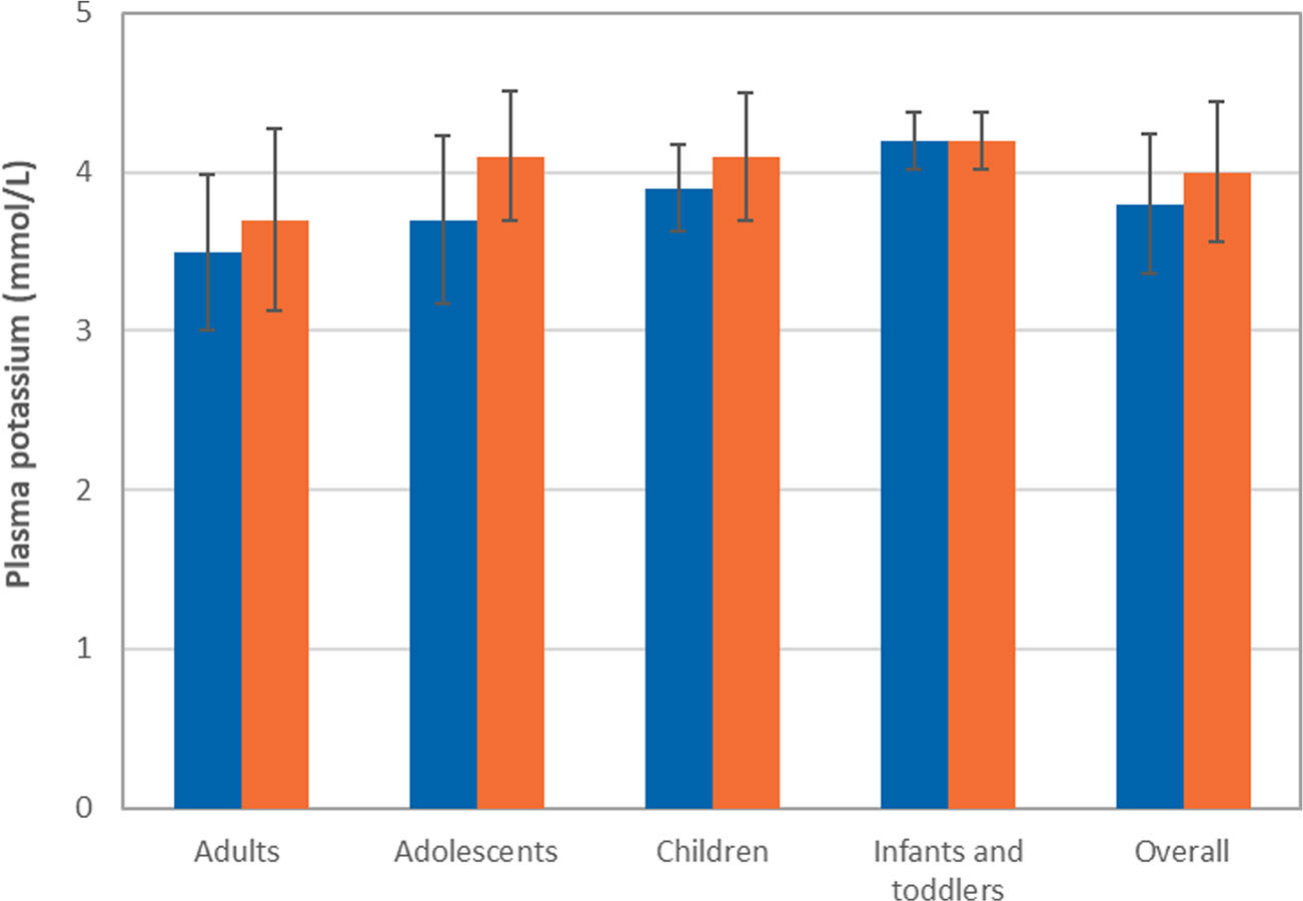
Steady state mean (± SD) plasma potassium levels in the different age groups before administration of the first morning dose of SoC (multiple daily intakes) and ADV7103 (morning and evening), ITT set. Blue bars: SoC, orange bars: ADV7103

The mean plasma potassium levels obtained during steady state treatment with SoC and ADV7103 are shown in Fig. 3, with overall mean ± SD values of 3.8 ± 0.4 under SoC and 4.0 ± 0.4 with ADV7103 in the ITT set (*n* = 34).

The response rate for potassium levels in the normal range (*n* = 29, ITT set) was high (82.8%) for both treatments and the same number/proportion of patients presented at least one episode of hypokalemia: 2 (6.9%) under SoC, 2 (6.9%) with ADV7103, and 3 (10%) during both treatment periods (Table 3).

**Table 3:** Contingency table showing the number (%) of responders for plasma potassium values and non-responders, presenting and at least one value of plasma potassium (mmol/L) below the normal lower limit, with SoC and ADV7103

Plasma potassium (ITT set, *n* = 29)		ADV7103	
		R	NR
SoC	R	22 (76%)	2 (6.9%)
	NR	2 (6.9%)	3 (10%)
*p* value		1.000	

*NR* non responders, *R* responders

### Urine citrate and urine calcium parameters

The numbers and proportions of responders and non-responders for urine parameters are shown in Table 4.

**Table 4:** Contingency tables showing the number (%) of responders for calciuria, citraturia, and urine calcium/citrate ratio and non-responders, presenting, respectively, at least one episode of UCa:UCr ratio (mmol/mmol) above the normal upper limit, at least one episode of UCi:UCr ratio (mmol/mmol) below the normal lower limit, and at least one value of UCa:UCi ratio (mmol/mmol) above the threshold considered for the risk of lithogenesis, with SoC and ADV7103

Calciuria			
UCa/UCr (ITT set, *n* = 30)		ADV7103	
		R	NR
SoC	R	27 (90%)	1 (3.3%)
	NR	1 (3.3%)	1 (3.3%)
*p* value		1.000	
Citraturia			
UCi/UCr (ITT set, *n* = 17)		ADV7103	
		R	NR
SoC	R	0 (0%)	1 (5.9%)
	NR	7 (41%)	9 (53%)
*p* value		0.070	
Urine calcium/citrate ratio			
UCa/UCi (ITT set, *n* = 20)		ADV7103	
		R	NR
SoC	R	3 (15%)	1 (5.0%)
	NR	9 (45%)	7 (35%)
*p* value		0.021*	

*NR* non responders, *R* responders

* Significant difference (according to McNemar’s test)

Hypocitraturia was observed in 16 (94.1%) of patients with SoC, while it was reported in only 10 (58.8%) of those under ADV7103 (*n* = 17, ITT set). A total of 7 (43.8%) of the 16 non-responders experiencing hypocitraturia with SoC became responders when switching to ADV7103, while among the 10 (58.8%) non-responders with ADV7103, only 1 (5.9%) was previously a responder under SoC. The difference between treatments did not reach statistical significance due to the limited number of patients in this analysis (*p* = 0.070).

No significant difference between treatments was observed for hypercalciuria, as evaluated by UCa/UCr ratio. Only 3 patients presented hypercalciuria, 1 with SoC, 1 under ADV7103, and 1 during both treatment periods.

Regarding UCa/UCi ratio, 9 (56.3%) of the 16 patients with values above the threshold for risk of lithogenesis with SoC had values below the threshold with ADV7103, while from the 8 patients above the threshold with ADV7103, only 1 was below the threshold with SoC. The difference between the proportions was statistically significant (McNemar’s *p* = 0.021, *n* = 20 evaluable patients, ITT set).

### Acceptability, tolerability, and safety

**Fig. 4:**
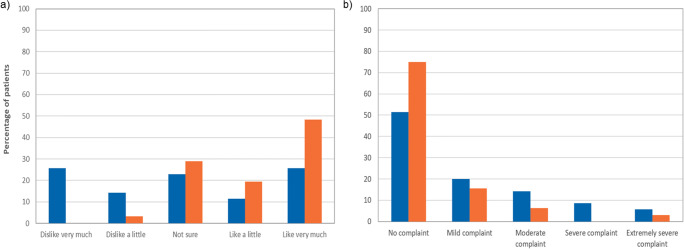
Percentage of patients for each **a** palatability and **b** gastrointestinal discomfort response category. Patient/parent-rated VAS or FHS scores translated into five categories. Blue bars: SoC, orange bars: ADV7103

Parameters of acceptability, such as ease of administration and ease of swallowing, were improved or preserved, under ADV7103 compared to SoC. Palatability was significantly improved with ADV7103 when compared to SoC, with an overall mean difference (95% CI) of 25 (10.7, 39.2) mm in the VAS. As shown in Fig. 4a, the proportion of patients who expressed some degree of dislike (very much or a little) was higher with SoC than with ADV7103: 40% (14/35) vs. 3.2% (1/31). Conversely, the proportion of patients who expressed a like for the taste of their medication (a little or very much) was higher with ADV7103 than with SoC: 67.7% (21/31) vs. 37.1% (13/35).

There were no AEs leading to drug discontinuation and no deaths reported during the study. Comparing steady state treatments, the proportion of patients who experienced at least one AE was similar between patients receiving ADV7103 (18.8%, 6/32) and those receiving SoC (18.9%, 7/37) but the proportion of patients with AEs considered as treatment-related was lower under ADV7103 (3.1%, 1/32) than under SoC (10.8%, 4/37).

The proportion of patients experiencing gastrointestinal AEs was lower during steady state treatment with ADV7103 than with SoC (3.1% vs. 13.5%). This was in agreement with significantly reduced gastrointestinal discomfort scores with ADV7103 when compared to SoC, with a mean difference (95% CI) of − 14.2 (− 25.9, − 2.6) mm in the VAS. The number of patients indicating any gastrointestinal discomfort was reduced by 50% under ADV7103 (25%, 8/32) as compared to SoC (48.6%, 17/35), as shown in Fig. 4b.

No safety issues were reported for laboratory parameters, physical examination, and vital signs. Only one child presented with an isolated and transient episode of mild hyperkalemia (4.7 mmol/L, for a normal range of 3.5–4.5 mmol/L according to the corresponding local laboratory) under ADV7103.

## Discussion

Current SoC treatments are immediate-release products and their effect is short-lived, due to prompt urinary clearance. Therefore, the treatment is given in several daily doses to compensate for short duration of action. Additionally, it often induces gastrointestinal side effects, probably due to a peak concentration of alkali load in the stomach, which may negatively impact adherence and overall efficacy.

For an optimized treatment with SoC, some patients require taking their medication during night-time. This is particularly the case for pediatric patients who are still growing, since chronic metabolic acidosis might impair growth velocity through IGF1 axis disturbances and bone quality through chronic buffer requirement [13].

Adequate metabolic control is essential for maintaining growth velocity and bone quality in pediatric patients with dRTA, and adequately treated children have been demonstrated to present significantly increased height values compared with those who are inadequately treated. It is also essential to limit nephrocalcinosis and nephrolithiasis, as well as the consequences of hypokalemia [25].

With only two daily administrations of ADV7103, plasma bicarbonate values were improved, with a more prominent benefit in pediatric patients, who presented particularly suboptimal values with SoC. The clinical benefit of ADV7103 is indicated by the reduction in the number of non-responders with plasma bicarbonate below the normal range when switching from SoC to the prolonged-release formulation. The rate of non-responders with current SoC treatments in our study is in agreement with that reported in a recent multicenter survey including data from 340 patients with dRTA, where only 57% of the patients showed plasma bicarbonate values ≥ 22 mmol/L [11]. In comparison, ADV7103 could offer a better metabolic control of the disease, according to the high percentage of patients (90%) ultimately presenting normal mean plasma bicarbonate values. The improved ability to correct metabolic acidosis with ADV7103 could be associated with the possibility of optimizing dosing due to its prolonged-release formulation, while poor tolerability and acceptability seem to limit further dosing increases with SoC. However, doses remain in agreement with the literature and among patients with dRTA, young children typically need higher doses than older children and adults [11].

Hypokalemia may persist in some patients despite sustained correction of systemic acidosis with SoC alkali therapy [26, 27] and these patients require potassium supplementation to maintain normal plasma potassium levels [28]. This justifies the treatment with potassium salts in ADV7103, which allows increasing plasma potassium without risk of hyperkalemia.

Potassium salts are preferred over sodium salts as the former have been shown to reduce urine calcium excretion, while the latter do not seem to affect urine calcium levels due to the effect of sodium on renal calcium reabsorption, although both increase urine citrate excretion levels [29].

Reduced urine citrate excretion and increased urine calcium excretion are contributing factors to stone-forming activity [25, 30, 31]. ADV7103 could also reduce the risk of stone formation in patients with dRTA by virtue of a better control of urine calcium and citrate excretion, as the difference between treatments in terms of urine calcium/citrate ratio was statistically significant in favor of the new treatment. In a previous study in healthy subjects, decreased urine calcium and increased urine citrate concentrations were observed with ADV7103 at doses ranging from 1 to 2.9 mEq/kg of alkali per day [18]. The present study confirms the increased urine citrate levels with ADV7103 in patients with dRTA, despite their need of buffers in blood, and almost half (44%) of non-responders for citraturia under SoC became responders under ADV7103. The percentage of patients with hypercalciuria was low (6.6%) with both treatments, as compared to 15% of patients with dRTA with reported hypercalciuria in an international patient cohort [11].

In conclusion, compared to SoC treatments, the prolonged-release combination of potassium citrate and potassium bicarbonate (ADV7103) significantly improves plasma bicarbonate levels and increases the response rate (from 43 to 90%) in patients with dRTA, while maintaining adequate plasma potassium levels. Normal levels of the urine calcium/citrate ratio obtained when switching to ADV7103 adds to the benefits of the prolonged-release formulation by reducing the factors inducing a risk of extensive nephrocalcinosis and stone formation. In addition to the control of metabolic acidosis, dosing scheme, acceptability, and gastrointestinal safety profiles are improved, bringing clinical benefit and supporting the use of ADV7103 as a first-line treatment for dRTA in adults and children over 6 months of age. Further evaluations are required to assess efficacy, safety, and adherence with ADV7103 in the long-term.

## Electronic supplementary material

ESM 1 (DOCX 57.6 kb)

ESM 2 (PPTX 62.3 kb)
