# Supplementary material for: Efficacy and safety of an innovative prolonged-release combination drug in patients with distal renal tubular acidosis: an open-label comparative trial versus standard of care treatments
Source: Pediatr Nephrol. 2020 Jul 26;36(1):83–91. doi: 10.1007/s00467-020-04693-2 (PMC7701073; doi:10.1007/s00467-020-04693-2)
Supplement: Supplementary file 1 — (DOCX 57.6 kb) [file 467_2020_4693_MOESM1_ESM.docx]

**Supplementary meterial**

**LABORATORY METHODS AND NORMAL RANGES: PLASMA**

# Abbreviations

d: days

m: months

w: weeks

y: years

M: male

F: female

Only ranges pertinent to study patients have been defined

# Laboratories related to site 001

| **Laboratory name/address** | **Parameters (units)** | **Normal ranges** | | **Analysis method** |
| --- | --- | --- | --- | --- |
| Biopole 21 Site du Parc de l’Europe  Dijon, FRANCE | Potassium (mmol/L) | Age: 3 y | 3.5 - 5.1 | Direct potentiometry |
|  | Bicarbonate (mmol/L) | Age: 3 y | 22 - 30 | Spectroreflectometry |
| Pôle de Biologie Hospice civils de Lyon (hospital Laboratory).  Lyon, FRANCE | Potassium (mmol/L) | Age:  28 d - 2 y  2 - 15 y  > 15 y | 4.1 - 5.3  3.4 - 4.7  3.5 - 4.8 | Indirect potentiometry  (Abbott Architect) |
|  | Bicarbonate (mmol/L) | Age:  28 d - 15 y  15 - 60 y  > 60 y | 20 - 28  22 - 29  23 - 31 | PEP carboxylase UV |

# Laboratories related to site 002

| **Laboratory name/address** | **Parameters (units)** | **Normal ranges** | | **Analysis method** |
| --- | --- | --- | --- | --- |
| Labosud OC Biologie  30300 Beaucaire, FRANCE | Potassium (mmol/L) | Age: 17 y | 3.5 - 5.1  Updated 10/03/2016:  3.4 – 5.1 | Indirect potentiometry  (Roche) |
|  | Bicarbonate (mmol/L) | Age: 17 y | 22 - 29 | PEP carboxylase UV |
| Pole Biologie CHU de Nîmes (hospital Laboratory).  Nîmes, FRANCE | Potassium (mmol/L) | Age:  1m - 3 y  3 -15 y  Adults | 4.1 - 5.3  3.4 - 4.7  3.5 - 5.1 | Potentiometry |
|  | Bicarbonate (mmol/L) | Adults | 22 - 29 | PEP carboxylase UV |

# Laboratories related to site 003

| **Laboratory name/address** | **Parameters (units)** | **Normal ranges** | | **Analysis method** |
| --- | --- | --- | --- | --- |
| Laboratoire de la Trillade (BIOTOP)  Avignon, FRANCE | Potassium (mmol/L) | Age: 6 - 8y | 3.4 - 4.5 | Indirect potentiometry |
|  | Bicarbonate (mmol/L) | Age: 6 - 8y | 22 - 28 | PEP carboxylase UV |
| Laboratoire Rey Port de Bouc  Port de Bouc, FRANCE | Potassium (mmol/L) | Age: 2 - 7 y | 3.5 - 5.0 | K Ion selective electrode (Beckman Coulter) |
|  | Bicarbonate (mmol/L) | Age: 2 - 7 y | 21 - 31 | CO_2_ ion selective electrode (Beckman Coulter) |
| Laboratoire Saint-André  Marseille, FRANCE | Potassium (mmol/L) | Age: 11 y | 3.5 - 4.5 | Ion selective electrode |
|  | Bicarbonate (mmol/L) | Age: 11 y | 20 - 28 | PEP carboxylase UV |
| Biologie médicale de l’APHM (hospital Laboratory).  Marseille, FRANCE | Potassium (mmol/L) | Age:  < 1 y  Adults | 3.7 - 5.2  3.4 - 4.5 | Indirect potentiometry |
|  | Bicarbonate (mmol/L) | Age:  1 - 6 m  Adults | 20 - 29  22 - 29 | PEP carboxylase UV |

# Laboratories related to site 005

| **Laboratory name/address** | **Parameters (units)** | **Normal ranges** | | | **Analysis method** |
| --- | --- | --- | --- | --- | --- |
| Laboratoire Stains Biolam - Clinique de l’Estrée  Stains, FRANCE | Potassium (mmol/L) | Age: 12 y | 3.4 - 4.6 | | Potentiometry (Roche) |
|  | Bicarbonate (mmol/L) | Age: 12 y | 20 - 29 | | PEP carboxylase UV |
| Laboratoire Astrabio des Flandres  Paris, FRANCE | Potassium (mEq/L) | Adults | | 3.4 - 4.5 | Indirect potentiometry |
|  | Bicarbonate (mEq/L) | Adults | | 21 - 31 | PEP carboxylase UV |
| Laboratoire de la gare Sartrouville  Sartrouville, FRANCE | Potassium (mmol/L) | Age: 3 y | 3.5 - 5.1 | | potentiometry |
|  | Bicarbonate (mmol/L) | Age: 3 y | 22- 29 | | PEP carboxylase UV |
| Laboratoire Bioplus Montereau  Montereau, FRANCE | Potassium (mmol/L) | Age: 8 y | 3.4 - 4.5 | | Ion selective electrode indirect potentiometry |
|  | Bicarbonate (mmol/L) | Age: 8 y | 22 - 29 | | PEP carboxylase UV |
| Laboratoire Caillault Bio 7.  Villeneuve-Saint-Georges, FRANCE | Potassium (mmol/L) | Age: 1 y | 4.1 - 5.3 | | Potentiometry |
|  | Bicarbonate mmol/L | Age: 1 y | 20 - 28 | | PEP carboxylase UV |
| Laboratoire de Biologie médicale Hopital Robert Debré (hospital laboratory).  Paris, FRANCE | Potassium (mmol/L) | Age:  1 m - 2 y  2 -15 y  > 15 y | 3.7 - 5.4  3.1 - 4.7  3.5 - 4.5 | | Direct potentiometry (Siemens on Advia 1800) |
|  | Bicarbonate (mmol/L) | Age :  1m - 2 y  2 - 15 y  > 15 y | 18 - 27  20 - 28  22 - 29 | | PEP carboxylase UV |

# Laboratories related to site 006

| **Laboratory name/address** | **Parameters (units)** | **Normal ranges** | | **Analysis method** |
| --- | --- | --- | --- | --- |
| Laboratoire Diaconat  Mulhouse, FRANCE | Potassium (mmol/L) | Age: Adults | 3.5 - 5.1 | Indirect Potentiometry |
|  | Bicarbonate (mmol/L) | Age: Adults | 21 - 32 | PEP carboxylase UV |
| Laboratoire de Biologie Médicale Service de Biochimie (hospital Laboratory)  Hôpital Louis Pasteur  Colmar, FRANCE | Potassium (mmol/L) | Age: >14 y | 3.5 - 5.1 | Indirect potentiometry |
|  | Bicarbonate (mmol/L) | Age: >14 y | 22 - 29 | PEP carboxylase UV |

# Laboratories related to sites 007 and 009

| **Laboratory name/address** | **Parameters (units)** | **Normal ranges** | | **Analysis method** |
| --- | --- | --- | --- | --- |
| Laboratoire CEF Boulard  Paris, FRANCE | Potassium (mmol/L) | Age: Adults | 3.4 - 4.5 | Ion selective electrode Beckman |
|  | Bicarbonate (mmol/L) | Age: Adults | 21 - 31 | PEP carboxylase UV (total C0_2_) |
| Laboratoire Lupescu Genevilliers  Gennevilliers, FRANCE | Potassium (mmol/L) | Age: 4 y | 3.4 - 4.5 | (heparin lithium takeoff) |
|  | Bicarbonate (mmol/L) | Age: 4 y | 21 - 31 | PEP carboxylase UV |
| Laboratoire du chemin de Fer  Lagny sur Marne, FRANCE | Potassium (mmol/L) | Age: > 18 y | 3.5 - 5.1 | Potentiometry  (Abbott Architect) |
|  | Bicarbonate (mmol/L) | Age: > 18 y | 22 - 29 | PEP carboxylase UV |
| Pole Biologie Médicale Hôpital Necker-enfants maladies (hospital Laboratory).  Paris, FRANCE | Potassium (mmol/L) | Age :  1 m - 13 y  Adults | 3.1 - 4.7  3.5 - 4.5 | Indirect potentiometry |
|  | Bicarbonate (mmol/L) | Age :  1 m - 3 y  3 - 15 y  Adults | 19 - 24  20 - 28  22 - 29 | PEP carboxylase UV |

# Laboratories related to site 008

| **Laboratory name/address** | **Parameters (units)** | **Normal ranges** | | **Analysis method** |
| --- | --- | --- | --- | --- |
| Laboratoire de la barrière de Toulouse Biopole 33  Bordeaux, FRANCE | Potassium (mmol/L) | Age: 4 y | 3.1 - 4.7 | Potentiometry Cobas 600 |
|  | Bicarbonate (mmol/L) | Age: 4 y | 22 - 29 | PEP carboxylase UV |
| Pôle Biologie du CHU de Bordeaux (hospital Laboratory).  Bordeaux, FRANCE | Potassium (mmol/L) | Age:  3m - 7 y  > 7 y | 3.5 - 5.3  3.5 – 5.0 | Indirect potentiometry |
|  | Bicarbonate (mmol/L) | 23 - 29 | | PEP carboxylase UV |

# Laboratories related to site 012

| **Laboratory name/address** | **Parameters (units)** | **Normal ranges** | | **Analysis method** |
| --- | --- | --- | --- | --- |
| Laboratoire du Près la Rose  Montbéliard, FRANCE | Potassium (mmol/L) | Age: > 18 y | 3.5 - 5.5 | Indirect potentiometry |
|  | Bicarbonate (mmol/L) | Age: > 18 y | 20 - 31 | PEP carboxylase UV |
| Laboratoire de Biologie Médicale CHRU de Besançon (hospital Laboratory).  Besançon, FRANCE | Potassium (mmol/L) | 3.5 - 5.1 | | Indirect potentiometry |
|  | Bicarbonate (mmol/L) | 21 - 32 | | PEP carboxylase UV |

#

# Laboratories related to site 013

| **Laboratory name/address** | **Parameters (units)** | **Normal ranges** | | **Analysis method** |
| --- | --- | --- | --- | --- |
| Laboratoire des Francs  Tourcoing, FRANCE | Potassium (mmol/L) | Age: 8 y | 3.5 - 5.0 | Indirect potentiometry |
|  | Bicarbonate (mmol/L) | Age: 8 y | 22 - 29 | PEP carboxylase UV |
| Laboratoire Clinique de l’esperance CHC  Montegnée, BELGIUM | Potassium (mmol/L) | Age: 3 y | 3.4 - 4.7 | *Not communicated* |
|  | Bicarbonate (mmol/L) | Age: 3 y | 22 - 26 | Indirect method with blood gas |
| Centre de Biologie CHRU de Lille  (hospital Laboratory).  Lille, FRANCE | Potassium (mmol/L) | Age:  1 - 12 m  1 - 18 y  > 18 y | 3.6 - 5.8  3.1 - 5.1  3.5 - 5.0 | Ion selective electrode Indirect potentiometry |
|  | Bicarbonate (mmol/L) | Age:  1 - 12 m  1 - 18 y  > 18 y | 17 - 29  20 - 31  22 - 29 | PEP carboxylase UV  (total CO_2_) |

# Laboratories related to site 015

| Laboratoire Bioliance  Saint Herblain, FRANCE | Potassium (mmol/L) | Age: 8 y | 3.7 - 5.2 | Indirect potentiometry |
| --- | --- | --- | --- | --- |
|  | Bicarbonate (mmol/L) | Age: 8 y | 22 - 29 | PEP carboxylase UV |
| Laboratoire Biologie Médicale Martineau - Desroys du Roure  La Roche sur Yon  FRANCE | Potassium (mmol/L) | Age: 12 y | 3.5 - 5.0 | Ion selective electrode |
|  | Bicarbonate (mmol/L) | Age: 12 y | 22 - 29 | PEP carboxylase UV |
| Laboratoire de biochimie Générale  Hotel Dieu. Centre Hospitalier Universitaire (hospital laboratory).  Nantes  FRANCE | Potassium (mmol/L) | Age:  1 - 24 m  2 - 12 y  12 - 120 y | 3.7 - 5.4  3.1 - 4.7  3.4 - 4.5 | Potentiometry |
|  | Bicarbonate (mmol/L) | Age:  3 - 52 w  1 - 2 y  2 - 12 y  12 - 120 y | 20 - 27  20 - 27  21 - 31  24 - 34 | PEP carboxylase UV |

#

# Laboratories related to siteS 202 AND 301

| **Laboratory name/address** | **Parameters (units)** | **Normal ranges** | **Analysis method** |
| --- | --- | --- | --- |
| Clinical Center of Nis  (hospital Laboratory)  Niš, SERBIA | Potassium (mmol/L) | 3.1 - 5.8 | Indirect potentiometry |
|  | Bicarbonate (mmol/L) | 18 - 25 | Indirect method with blood gas |
| Children’s University Hospital Bratislava (hospital laboratory).  Bratislava, SLOVAKIA | Potassium (mmol/L) | 3.5 - 5.5 | Ion selective electrodes, indirect method (Roche) |
|  | Bicarbonate (mmol/L) | 22 - 26 | Calculated using Hendersen-Hasselbalch equation: Derived parameter from RapidLab 1265 (Siemens) |

**NORMAL RANGES: URINE RATIOS AND RISK OF LITHOGENESIS**

| **Urine parameters (units)** | **Normal values** | | |
| --- | --- | --- | --- |
| *Urine ratios* | | | |
| Calcium/creatinine (mol/mol) [1, 2] | 5 to 11 m  1 to < 2 y  2 to < 3 y  3 to < 5 y  5 to < 7 y  7 to < 10 y  10 to < 14 y  14 to 17 y  Adults | ≤ 2.2  ≤ 1.5  ≤ 1.4  ≤ 1.1  ≤ 0.8  ≤ 0.7  ≤ 0.7  ≤ 0.7  ≤ 0.5 | |
| Citrate/creatinine (mmol/mmol) [3] | M 2 to <7 y  M 7 to <13 y  M 13 to <18 y  M Adults  F 2 to <7 y  F 7 to <13 y  F 13 to <18 y  F Adults | ≥ 0.142  ≥ 0.082  ≥ 0.052  ≥ 0.052*  ≥ 0.171  ≥ 0.154  ≥ 0.127  ≥ 0.127* | |
| *Risk of lithogenesis* | | | |
| Calcium/citrate (mmol/mmol) [4] | All | | ≤ 3 |

* The reference limit for adolescents also applied to adults according to expert opinion

1. Frey J, Daudon M, Raby N et al (2001) Valeur sémiologique des paramètres biochimiques urinaires. Ann Biol Clin 59:13–25

2. Matos V, van Melle G, Boulat O et al (1997) Urinary phosphate/creatinine, calcium/creatinine, and magnesium/creatinine ratios in a healthy pediatric population. J Pediatr 131:252–257

3. Kirejczyk JK, Porowski T, Konstantynowicz J et al (2014) Urinary citrate excretion in healthy children depends on age and gender. Pediatr Nephrol 29:1575–1582

4. Parent X, Boess G, Brignon P (1999) Lithiase oxalocalcique. Relation entre facteurs de risque biochimiques et phase cristalline du calcul. Prog Urol 9:1051–1056
