# Supplementary material for: Efficacy and safety of an innovative prolonged-release combination drug in patients with distal renal tubular acidosis: an open-label comparative trial versus standard of care treatments
Source: Pediatr Nephrol. 2020 Jul 26;36(1):83–91. doi: 10.1007/s00467-020-04693-2 (PMC7701073; doi:10.1007/s00467-020-04693-2)
Supplement: Supplementary file 2 — (PPTX 62.3 kb) [file 467_2020_4693_MOESM2_ESM.pptx]

## Slide 1
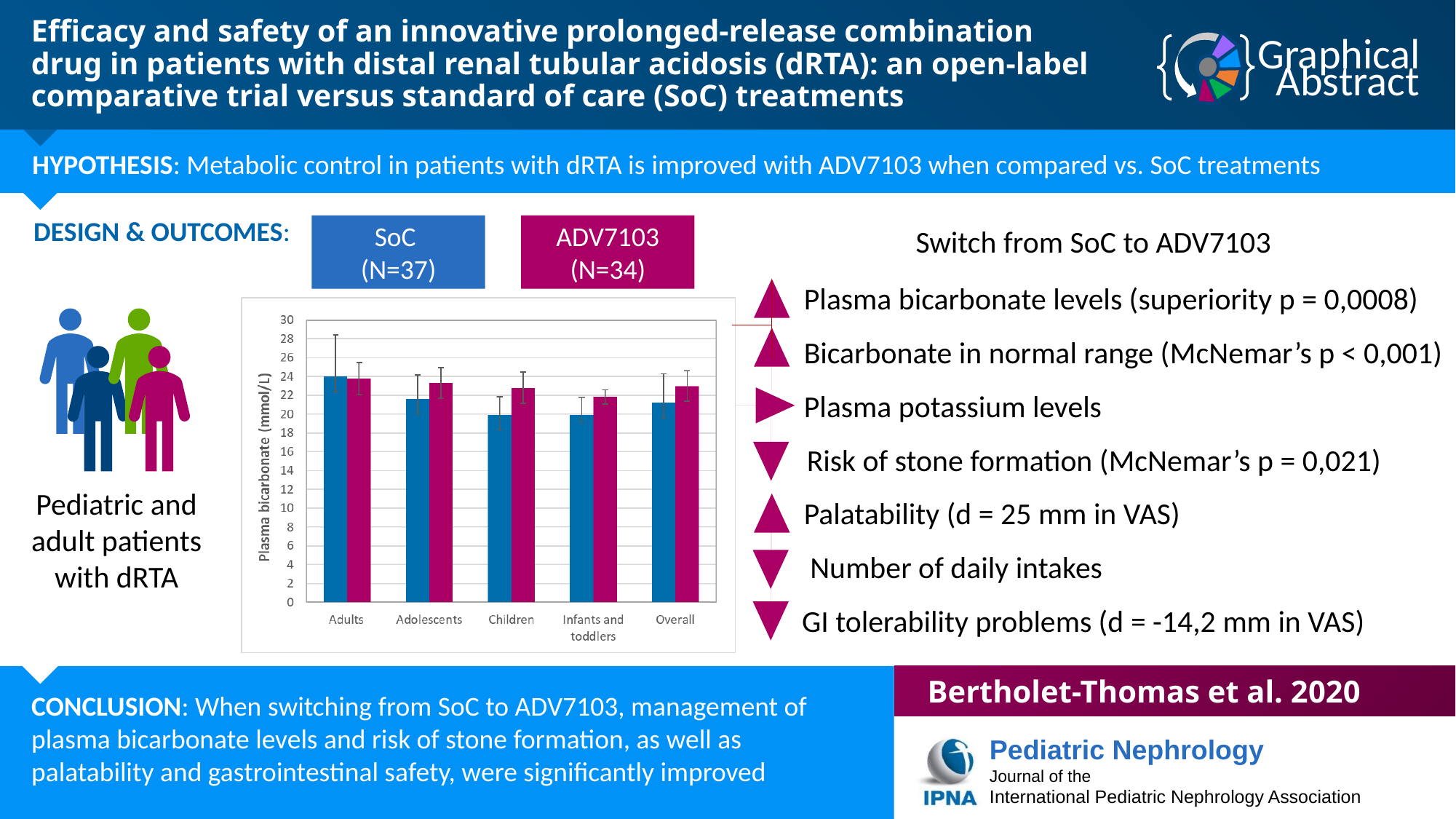

Efficacy and safety of an innovative prolonged-release combination drug in patients with distal renal tubular acidosis (dRTA): an open-label comparative trial versus standard of care (SoC) treatments
HYPOTHESIS: Metabolic control in patients with dRTA is improved with ADV7103 when compared vs. SoC treatments
DESIGN & OUTCOMES:
SoC
(N=37)
ADV7103 (N=34)
Switch from SoC to ADV7103
Plasma bicarbonate levels (superiority p = 0,0008)
Bicarbonate in normal range (McNemar’s p < 0,001)
Plasma potassium levels
Risk of stone formation (McNemar’s p = 0,021)
Pediatric and adult patients with dRTA
Palatability (d = 25 mm in VAS)
Number of daily intakes
GI tolerability problems (d = -14,2 mm in VAS)
Bertholet-Thomas et al. 2020
CONCLUSION: When switching from SoC to ADV7103, management of plasma bicarbonate levels and risk of stone formation, as well as palatability and gastrointestinal safety, were significantly improved
